# Secretase inhibition in Alzheimer’s disease therapeutics reveals functional roles of amyloid-beta42

**DOI:** 10.4103/NRR.NRR-D-24-01481

**Published:** 2025-04-29

**Authors:** Timothy Daly, Bruno P. Imbimbo

**Affiliations:** UMR 1219 (Bordeaux Population Health) University of Bordeaux & INSERM, Bordeaux, France; CNS UMR 5164 (ImmunoConcept), University of Bordeaux, Bordeaux, France; Bioethics Program, FLACSO Argentina, Tucumán, Buenos Aires, Argentina; Research & Development, Chiesi Farmaceutici, Parma, Italy

In the words of the late Sir Colin Blakemore, neurologists have historically sought to infer brain functions in a manner akin to taking a hammer to a computer—analyzing localized anatomical lesions caused by trauma, tumors, or strokes, noting deficits, and inferring what functions certain brain regions may be responsible for. This approach exemplifies a deletion heuristic, where the absence of a specific function reveals insights about the underlying structures or mechanisms responsible for it. By observing what is lost when a particular brain region is damaged, throughout the history of the field, neurologists have pieced together the intricate relationship between anatomy and function.

Over time, this heuristic approach has significantly evolved. Originally grounded in gross anatomical post-mortem observations in the 19^th^ and early 20^th^ centuries, it has advanced considerably with new methodologies. The emergence of neuroimaging technologies, such as MRI and PET, enabled the precise localization of lesions and their functional implications in living patients. This progress further facilitated the integration of neurogenetics, where the identification of genetic mutations and their effects on neural function shed light on the molecular mechanisms underlying neurological disorders, extending to protein function. Today, this expanded process remains central to neuroscience, illustrating how traditional ways of thinking continue to form a crucial foundation, even as neurology advances toward increasingly precise molecular methodologies. A similar approach can be applied to Alzheimer’s disease (AD), where specific protein dysfunctions, particularly those involving amyloid-β42 (Aβ_42_) and tau, are key contributors.

**Figure 1:**
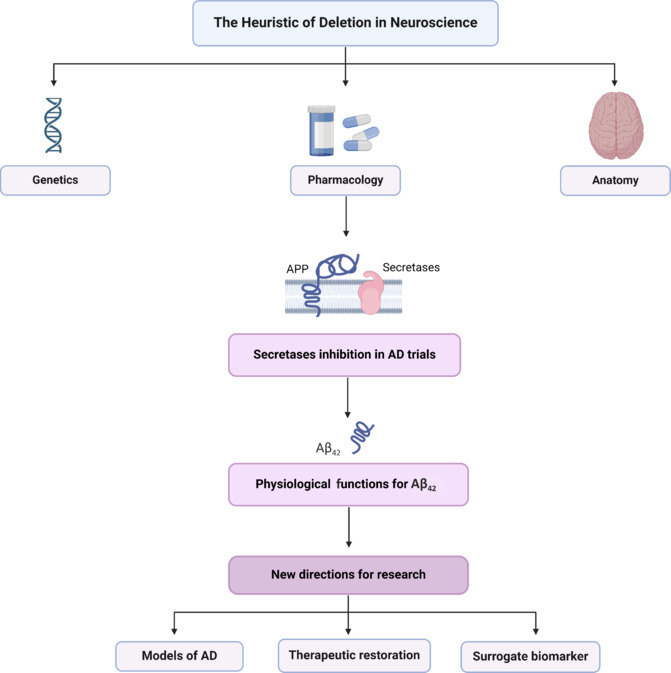
The history of neurology shows that neuroscientists often work backward to infer function from anatomical and genetic deletions. The pharmacological inhibition of enzymes in trials of AD responsible for producing Aβ_42_ represents a functional pharmacological lesion that could inform our understanding of the hypothetical role of Aβ_42_ in maintaining cognitive and neuronal functionality in AD. This challenges the prevailing Aβ-lowering therapeutic strategy, prompts a reevaluation of how we approach the biology of Aβ_42_ in the context of AD and suggests new directions for research. Created with BioRender.com. Aβ_42_: Amyloid-β42; AD: Alzheimer’s disease; APP: amyloid precursor protein.

**Enzyme inhibition – a functional pharmacological depletion in AD therapeutics:** We apply the deletion heuristic to interpret the effects of drug-induced inhibition of enzyme function observed in clinical trials of AD, particularly with inhibitors of β-secretase, the enzyme responsible for producing Aβ_42_ from its precursor, amyloid precursor protein. These pharmacological interventions, designed to reduce the production of Aβ_42_, offer a functional perspective on its role in the disease (**Figure 1**). Strikingly, these trials have revealed that Aβ_42_, often viewed as exclusively pathogenic, appears to have a functional role in promoting cognitive and neuronal performance in AD. This finding aligns with recent, seemingly counterintuitive observations that approved anti-amyloid antibodies, such as lecanemab and aducanumab, increase levels of soluble Aβ_42_ in the cerebrospinal fluid (CSF), likely through liberation from plaque deposits (Abanto et al., 2024).

Conversely, the depletion or removal of Aβ_42_ has been shown to exacerbate cognitive decline and increase mortality in AD patients (Panza et al., 2019). These findings suggest that while the excess deposition of Aβ_42_ into plaques may be neurotoxic, its soluble monomeric form likely plays some physiological and/or neuroprotective role, highlighting the complexity of targeting this molecule in therapeutic strategies (Imbimbo et al., 2023). Such evidence challenges the conventional “one-size-fits-all” approach to amyloid-targeting therapies and underscores the importance of carefully balancing the reduction of pathological amyloid deposition with the preservation of its functional, soluble forms. This nuanced understanding demonstrates the value of the deletion heuristic in elucidating both detrimental and beneficial roles of biological molecules in complex diseases such as AD.

In AD trials, β-secretase inhibitors significantly worsen cognitive performance. In a 78-week, double-blind, placebo-controlled trial of the β-secretase inhibitor verubecestat in 1958 participants with mild-to-moderate AD, the drug dose-dependently lowered Aβ_42_ concentrations (up to 84%) in CSF, with patients showing no cognitive or functional benefit and increased toxicity, leading to early termination (Egan et al., 2018). Mortality rates in the placebo, 12 mg/day, and 40 mg/day treatment groups were 0.8%, 1.4%, and 1.8%, respectively (logistic regression; *r*² = 0.833, one-tail *P* = 0.056). Post-trial, many patients commenced open-label treatment: 360 placebo recipients received 40 mg/day, while 346 12 mg recipients and 333 40 mg recipients continued their starting dose. During the shortened post-trial period, 4 deaths occurred in the 12 mg group and 10 deaths in the 40 mg group (8 former placebo; 2 former 40 mg). Mortality among the original placebo recipients increased from 5/653 (0.8%) to 8/360 (2.2%) on 40 mg verubecestat (two-tail *P* = 0.049). These safety data are consistent with other controlled studies of β-secretase inhibitors in AD (Imbimbo et al., 2019). Moreover, data from semagacestat, a γ-secretase inhibitor that also reduces Aβ_42_ production, confirm the worsening of cognition in AD patients (Doody et al., 2013).

These observations support the hypothesis that Aβ_42_ is essential for cognition and functionality in AD patients. The detrimental effects of β-secretase and γ-secretase inhibitors on cognition are likely attributable to the reduced production of Aβ_42_ from amyloid precursor protein. This is consistent with a recent meta-analysis of nearly 26,000 AD patients across 24 long-term, placebo-controlled anti-Aβ clinical trials, which identified a highly significant positive association between CSF Aβ_42_ levels and both cognitive and clinical performance. These findings help explain the therapeutic relevance of the paradoxical increase in Aβ_42_ levels observed with anti-Aβ antibodies, including the Food and Drug Administration-approved lecanemab (Abanto et al., 2024).

However, the dominant amyloid hypothesis posits that Aβ_42_ is inherently neurotoxic, and its accumulation drives the progression of AD. Proponents of this view attribute the observed cognitive worsening in patients treated with β- and γ-secretase inhibitors to off-target toxicity rather than to the inhibition of Aβ_42_ production itself. This explanation, however, introduces a paradox: if the off-target effects of these drugs are responsible for the decline in cognition, it implies that the off-target pathways have a more critical role in regulating cognitive function than Aβ_42_, thereby undermining the central claim of the amyloid hypothesis—that Aβ deposition is the cause of cognitive decline in AD.

Instead, an alternative perspective emerges when viewing the pharmacological inhibition of β- and γ-secretases as analogous to traditional lesion studies in neuroanatomy or neurogenetics. Just as the deletion of specific brain regions or genetic mutations can elucidate their physiological roles, the pharmacological inhibition of the enzymes responsible for producing Aβ_42_ acts as a functional lesion. This approach has provided valuable insights, revealing that Aβ_42_ may play a significant role in maintaining cognitive and neuronal functionality in AD. Such findings challenge the notion of Aβ_42_ as merely neurotoxic and instead suggest a dual role, with its soluble, monomeric form potentially contributing to synaptic and neuronal support.

This reevaluation casts doubt on the prevailing therapeutic strategy focused on broadly lowering Aβ_42_ levels. While reducing the pathological deposition of Aβ_42_ in plaques has provided some beneficial effects, growing evidence suggests that this approach may inadvertently disrupt its physiological roles. Soluble monomeric Aβ_42_ has been implicated in synaptic plasticity, neuronal activity, and memory processes, indicating that its presence may be essential for maintaining normal brain function (reviewed in Espay et al., 2023). This duality raises critical questions about the potential unintended consequences of overly aggressive amyloid-targeting therapies, including exacerbation of cognitive decline and increased mortality, as previously mentioned (Panza et al., 2019). By reframing pharmacological interventions as tools to study and understand complex functions of Aβ_42_, rather than solely as treatments aimed at its elimination, researchers can adopt a more holistic approach to AD therapy. This perspective emphasizes the importance of preserving the physiological benefits of soluble Aβ_42_ while simultaneously addressing its pathological aggregation into plaques. Indeed, recent studies have shown that anti-Aβ monoclonal antibodies can mobilize amyloid from plaques, potentially increasing the interstitial pool of soluble Aβ_42_, which may positively influence neuronal function (Abanto et al., 2024). These findings underscore the complexity of targeting Aβ_42_ and the necessity of developing therapies that achieve a delicate balance between mitigating its toxic effects and preserving its beneficial roles.

**Restoring the physiological function of Aβ**_**42**_ **in AD:** The loss of normal protein homeostasis is a well-recognized hallmark of aging, which is the most significant risk factor for AD. However, from the outset of protein-centric thinking in AD, the dominant perspective has focused almost exclusively on the therapeutic hypothesis of a toxic gain of function via Aβ assembly and deposition. In contrast, we argue that the insights from the previous discussion on pharmacological lesions, coupled with extensive evidence supporting the normal physiological functions of Aβ—including its role in regulating synaptic activity and the association of low Aβ_42_ levels with human AD pathology (Espay et al., 2023)—should shift the focus toward counterbalancing the loss of Aβ_42_, or “proteinopenia,” as a therapeutic strategy (Espay et al., 2023).

Various pharmacological approaches exist to restore Aβ_42_ levels. Paradoxically, as previously noted, recently approved anti-Aβ antibodies increase soluble Aβ_42_ (Abanto et al., 2024), likely by liberating it from amyloid plaques. However, this is not a recommended strategy for Aβ_42_ restoration, as these antibodies are specifically designed to achieve the opposite effect—reducing amyloid burden. A more promising pharmacological approach might involve a class of molecules known as “aftins” (Aβ_42_ inducers), which enhance β-secretase and γ-secretase activity, leading to increased production of Aβ_42_ (Meunier et al., 2015). Originally designed to model AD pharmacologically based on the assumption that Aβ_42_ accumulation is toxic (Hochard et al., 2013), aftins have provided preliminary and conflicting insights into the effects of increasing Aβ_42_. In wild-type mice, increasing Aβ_42_ beyond physiological levels with aftin-4 may be neurotoxic, according to one 2015 study (Meunier et al., 2015), or non-toxic according to a study from 2024 (Žnidaršič et al., 2024). Aftin-5 (also known as PDD005), was shown to strongly upregulate Aβ_42_ (with an increase of over 900%) (Hochard et al., 2013) and, in aged mice, it rescued cognitive deficits, prevented neurogenesis impairment, enhanced synaptic function, and reduced neuroinflammation (Guyot et al., 2020). Further studies on aftins are warranted and require appropriate preclinical disease models.

PS1 and PS2 conditional double knockout (cDKO) mice exhibit a range of AD-like phenotypes, including cortical and hippocampal atrophy, glial cell proliferation, neuronal loss, neurofibrillary tangles, and age-related cognitive impairment (Feng et al., 2004). However, cDKO mice do not develop Aβ deposition in the brain with age, but rather display altered Aβ_42_ levels (Feng et al., 2004). Interestingly, Duan et al. (2022) demonstrated that exogenous synthetic Aβ_42_ monomers improve memory deficits not only in cDKO mice, which lack Aβ_42_ deposition, but also in amyloid precursor protein/PS1/Tau triple-transgenic 3×Tg-AD mice, soluble Aβ_42_ in CSF also decreases in 3 × Tg-AD mice with age (Cho et al., 2016). This finding highlights the potential neuroprotective role of soluble Aβ_42_ monomers in restoring cognitive function, even in the context of AD-like pathology. Furthermore, this preclinical model offers a robust platform for testing the safety and efficacy of Aβ_42_ restoration as a therapeutic approach, potentially paving the way for human trials.

Beyond aftins, repurposed drugs present another promising avenue. Metformin, a widely prescribed anti-diabetic medication, has been associated with a dose-dependent reduced risk of AD. Emerging evidence suggests that metformin may increase soluble Aβ_42_ levels through enhanced secretase activity, making it a potential candidate for Aβ_42_ restoration (Daly and Imbimbo, 2025). Such approaches not only offer hope for mitigating AD progression but also underscore the need for change in AD research—moving from the exclusive reduction of amyloid burden toward restoring the physiological balance of Aβ_42_ for cognitive and neuronal health.

**Conclusions:** The pharmacological inhibition of enzymes responsible for producing Aβ_42_ represents a functional pharmacological lesion that could inform our understanding of the hypothetical role of Aβ_42_ in maintaining cognitive and neuronal functionality in AD. This challenges the prevailing Aβ-lowering therapeutic strategy and prompts a reevaluation of how we approach the biology of Aβ_42_ in the context of AD. From these insights, three major actionable conclusions emerge, each with potential implications for future research and treatment strategies.

The first conclusion emphasizes the need for further research into the effects of sub-physiological levels of Aβ_42_ in AD. Experimental models, such as cDKO mice, provide valuable tools for investigating how reduced CSF Aβ_42_ levels impact neuronal function and cognitive performance, offering insights into its physiological role (Duan et al., 2022). In their study, Duan et al. (2022) observed that physiological concentrations of Aβ_42_ monomers did not affect the basal level of synaptic transmitter release, as measured by paired-pulse facilitation. However, Aβ_42_ monomers significantly influenced presynaptic transmitter release during tetanic stimulation, as reflected in post-tetanic potentiation. Post-tetanic potentiation refers to the enhanced release of synaptic transmitters mediated by a transient increase in Ca²⁺ concentration within presynaptic terminals during tetanic stimulation. This finding suggests that Aβ_42_ is involved in mechanisms of synaptic plasticity only when associated with high-frequency stimulation, where Ca²⁺ levels in presynaptic terminals surpass a critical threshold. Conversely, Aβ_42_ appears to have no effect during low-frequency stimulation, where Ca²⁺ buildup is insufficient to activate these mechanisms. Moreover, this indicates that the role of Aβ_42_ in enhancing glutamate release is closely tied to Ca²⁺ dynamics within presynaptic terminals. These insights may help clarify the dual roles of Aβ_42_—highlighting its toxic effects at elevated levels versus its functional necessity at other more appropriate physiological concentrations. Continued research in this area could lead to more nuanced therapeutic strategies aimed at preserving physiological roles of Aβ_42_ while mitigating its pathological effects.

The second conclusion involves the methodological generalization of the meta-analytic approach employed by Abanto et al. (2024). Their findings highlight the critical relationship between cognitive performance and CSF Aβ_42_ levels, suggesting that the restoration of Aβ_42_ to physiological levels could serve as a methodological outcome or surrogate marker in studies of pharmacological and non-pharmacological interventions for AD. Incorporating Aβ_42_ restoration as a standard metric could provide a unifying framework for evaluating diverse treatment modalities, including lifestyle interventions and novel drug therapies.

The third conclusion calls for the direct testing of the therapeutic hypothesis that Aβ_42_ restoration may benefit AD patients. This could involve innovative therapeutic approaches, such as aftins, which selectively enhance Aβ_42_ production, or the repurposing of existing drugs like metformin, which has shown promise in modulating Aβ dynamics and metabolic pathways implicated in AD. These strategies represent a shift from simply targeting Aβ_42_ for reduction to leveraging its physiological properties for therapeutic benefit, potentially opening new avenues for disease modification.

Taken together, these conclusions advocate for new priorities in AD research and treatment, emphasizing the need to better understand and harness the potential physiological roles of Aβ_42_ while refining therapeutic strategies to balance beneficial and pathological effects. This approach could lead to effective and nuanced interventions for AD, but will require further direct tests of the therapeutic value of the restoration of Aβ_42_.

*Dr. Timothy Daly has no financial relationships or conflicts of interest to declare. Dr. Bruno P. Imbimbo is an employee at Chiesi Farmaceutici. He is listed as an inventor in a number of Chiesi Farmaceutici’s patents of anti-Alzheimer drugs. No conflicts of interest exist between Chiesi Farmaceutici and publication of this article*.

*The words of Sir Colin Blakemore were paraphrased from a 2014 public lecture he gave at the “Brain Matters” event at the University of Nottingham, attended by Timothy Daly (https://www.nottingham.ac.uk/research/groups/neuroscience/brainmatters/an-evening-with/tickets.aspx#book)*.
